# Cervical Cancer Mortality in Romania: Trends, Regional and Rural–Urban Inequalities, and Policy Implications

**DOI:** 10.3390/medicina58010018

**Published:** 2021-12-23

**Authors:** Florentina Furtunescu, Roxana Elena Bohiltea, Adrian Neacsu, Corina Grigoriu, Corina Silvia Pop, Nicolae Bacalbasa, Ionita Ducu, Ana-Maria Iordache, Radu Virgil Costea

**Affiliations:** 1 Department of Public Health and Management, Faculty of Medicine, “Carol Davila” University of Medicine and Pharmacy Bucharest, 050463 Bucharest, Romania; florentina.furtunescu@umfcd.ro; 2 Department of Obstetrics and Gynecology, “Carol Davila” University of Medicine and Pharmacy Bucharest, 020021 Bucharest, Romania; adrianneacsu2006@yahoo.com (A.N.); corigri@gmail.com (C.G.); or nicolaebacalbasa@gmail.com (N.B.); 3 Department of Obstetrics and Gynecology, “Sfantul Ioan” Emergency Clinical Hospital, 042122 Bucharest, Romania; 4 Department of Obstetrics and Gynecology, University Emergency Hospital Bucharest, 050098 Bucharest, Romania; ionitaducu@gmail.com; 5 Department of Internal Medicine and Gastroenterology, “Carol Davila” University of Medicine and Pharmacy Bucharest, 020021 Bucharest, Romania; cora.pop@gmail.com; 6 Department of Internal Medicine and Gastroenterology, University Emergency Hospital Bucharest, 050098 Bucharest, Romania; 7 Optospintronics Department, National Institute for Research and Development in Optoelectronics-INOE 2000, 409 Atomistilor, 077125 Magurele, Romania; 8 Department of Surgery, “Carol Davila” University of Medicine and Pharmacy Bucharest, 020021 Bucharest, Romania; rcostea2000@yahoo.com; 9 Department of Surgery, University Emergency Hospital Bucharest, 050098 Bucharest, Romania

**Keywords:** cervical cancer deaths, age-standardized mortality, cervical cancer screening, cervical cancer mortality

## Abstract

*Background and Objectives:* Despite being largely preventable, cervical cancer mortality still remains an important public health problem globally, in Europe, and in Romania. The European Union member states are urged to implement systematic, population-based screenings for cervical cancer, but the programs developed by the countries remain very heterogeneous. This study aimed to investigate the differences in cervix cancer mortality between Romania and EU and within Romania over the last two decades and to reveal the major sources of inequalities and the policy implications. *Materials and Methods:* We analyzed the number of deaths and the mortality rates by cervical cancer, standardized using the direct method, over two decades (2001–2016 for the EU, and 2001–2019 for the national and sub-national analyses). Trends, mortality reduction over the years, and mortality differences at the beginning and end of the time interval have been calculated for the EU and Romania, at national and sub-national levels (rural–urban and regions). *Results:* Our results revealed differences in cervical cancer mortality between Romania and EU and within Romania (among regions and rural–urban areas). These differences used to be very high in the past and are still persisting. *Conclusions:* The country should revisit its national cervical cancer screening program, which has been implemented for many years, but with a very limited participation rate. Due to the similar problems existing in Central-Eastern Europe, targeted support from the EU for the members from this geographical area could contribute to the minimization of differences in cervical cancer mortality among the EU members.

## 1. Introduction

Cervical cancer mortality is largely preventable, through vaccination against Human Papiloma Virus (HPV), citology- or HPV-based cancer screening, treatment of precancerous lesions, and improved access to diagnosis and treatment of the invasive cancers [1,2,3]. Despite this potential of preventability, cervix cancer still remains an important public health problem globally, with an estimate of 569,847 new cases and 311,365 deaths per year in 2018 [4]. From around 52% of cases, 60% of the deaths occur in low- and middle-income counties due to failure in implementing population-based preventive programs [4,5,6]. In Europe, cervix cancer is responsible for an estimate of 58,169 new cases, with 25,989 just in 2020 [7].

In the European Union (EU), a Recommendation of the Council since 2003 urges the member states to implement systematic, population-based cancer screenings for breast, cervix, and colon cancer [8]. In the case of cervical cancer, conventional cytology for cancer precursors with Papanicolau staining, validated liquid-based cytology, primary testing for oncogenic HPV with validated assays, and implementation of HPV vaccination programs have been recommended [9,10,11]. The last assessment of this recommendation revealed that 22 (out of 28) member states had implemented national or sub-national cervix cancer screening programs, ensuring an average coverage of 59.2% women aged 30–59 years, a participation rate of 50.7%, and an examination coverage of 29.8% [12]. Additionally, the screening programs developed by the countries were marked by wide heterogeneity, which made it difficult to compare the quality of the assurance measures, the monitoring and evaluation strategies, or the cost-effectiveness [12,13,14]. Still, cervix cancer was responsible for 9744 deaths in 2016 (last available year), with wide disparities in mortality rates still persisting among the countries (e.g., ten times variations between Italy and Romania, from 0.71 to 8.04 deaths per 10,000 women, standardized rates) and within the country itself [15].

Beyond the high number of deaths, cervical cancer has multiple effects on the health status, by affecting the quality of life, sexual health, and, due to its predilection in young women, by threatening fertility [16,17]. Even in the case of successful treatment of the precancerous lesions and preservation of fertility, it induces a risk of preterm birth [18,19,20].

Our study aimed to investigate the differences in cervical cancer mortality between Romania and the EU and within Romania over the last two decades and to reveal the major sources of inequalities and the policy implications for better control of this public health problem in the future.

## 2. Materials and Methods

We followed the differences in cervical cancer mortality from two perspectives—differences between Romania and EU and differences within the country itself, by regions, and rural–urban areas. We used the number of deaths and the mortality rates by cervical cancer—Code C53 upon the International Classification of Disease Revision 10 (ICD-10), which is currently in use in the country [21]. The time interval for the analysis was 2001–2016, for the comparison to EU average (2016 being the last available year for the EU average) and 2001–2019 for the national data.

**Figure 1:**
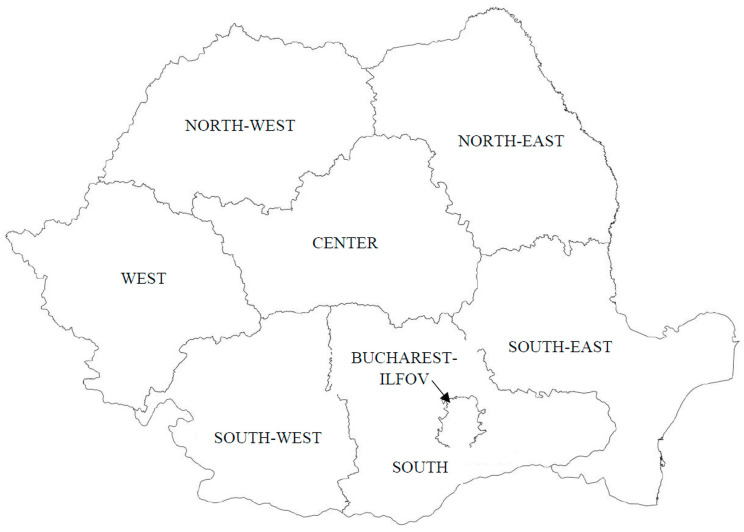
Romania—the geographic map by regions.

The geographic area: for EU, we used the EU 27 data (the United Kingdom excluded). For Romania, we used data disaggregated by rural–urban area and by NUT 2 region (Nomenclature of territorial units for statistics for basic regions). Romania is the eighth largest EU country by surface and the sixth country by population (22.1 million citizens registered, among which 19.3 million inhabitants reside in the country) [22]. Currently, 46% of the total population lives in rural areas, this being the highest proportion of rural population among all the EU member states [23]. The country is divided into 42 counties and 8 regions NUTS 2: North-East (NE), South-East (SE), South (S), South-West (SW), West, North-West (NW), Center (C), and Bucharest–Ilfov (BI), last one being the most developed and including the capital city (Figure 1).

Deaths analysis: The deaths have been analyzed as annual number and percentage change in 2016/2019, compared to the baseline (2001).

Mortality rates analysis: For the comparison of Romania versus EU, we used the standardized mortality rates extracted from Eurostat [15]. For the further sub-national analysis, we have standardized the national and sub-national data using the direct method (standard population) [24]. We used the following data: (i) the number of deaths by cervical cancer, disaggregated by five years’ age groups, region, and rural–urban areas [25]; (ii) the female population by five-year age-groups, per country, per region, and per rural–urban population [22]. Our standard population was represented by a national female population calculated for each five-year age group as the arithmetic mean of corresponding age group female population for years 2001, 2010, and 2019 (first, last, and middle year of the study interval).

We followed the trends in standardized mortality rate (SMR), mortality reduction (MR), and mortality difference (MD).

The mortality reduction (MR)

The mortality reduction has been calculated in EU, per country, and for the sub-national levels in the last year of the time interval, compared to the starting year. For the EU and country levels, we used the formula:

MR_A_(%) = (SMR_A,2001_ − SMR_A,2016_)/SMR_A,2001_ (1)

where:

- MR_A_(%) is the mortality reduction in area A, in 2016 compared to 2001, expressed as percentage;
- Area A is either Romania or EU;
- SMR_A_ is the standardized mortality rate in geographical area A.

For the subnational levels, we calculated MRs for urban national, rural national, regionals, urban regionals, and rural regionals SMRs, by using the following formula:

MR_B_(%) = (SMR_B,2001_ − SMR_B,2019_)/SMR_RO,2001_ (2)

where:

- MR_B_(%) is the mortality reduction in area B, in 2019, expressed as percentage from the national SMR for year 2001;
- Area B could be urban national, rural national, regional, or urban/rural regional level;
- SMR_B_ is the standardized mortality rate in geographical area B, for the corresponding year (2001 or 2019);
- SMR_RO,2001_ is the standardized mortality rate in Romania, in 2001. We have chosen to express all the reductions as proportion from the national rate, for a unique baseline, which is the national model.

The mortality difference (MD)

It has been calculated as annual difference between Romania and EU SMRs or between national and sub-national levels. We explored the differences at the beginning and, respectively, the end of the time interval (2001 and 2016 for Romania and EU, and 2001 and 2019 within the country) by using the formulas:

MD_RO, EU,i_(%) = (SMR_RO, year i_ − SMR_EU,i_)/SMR_EU,i_ (3)

where:

- MD_A,EU,i_ is the mortality difference between Romania and EU in year i, expressed as percentage;
- Year i is either 2001 or 2016;
- SMR_RO,i_ is the standardized mortality rate in Romania, for the year i;
- SMR_EU,i_ is the standardized mortality rate in EU, for the year i.

MD_B,j_(%) = (SMR_B,j_ − SMR_RO,j_)/SMR_RO,j_ (4)

where:

- MD_B,j_ is the mortality difference between area B and Romania in year j, expressed as percentage.
- Area B could be any subnational area, like urban national, rural national, or region.
- Year j is either 2001 or 2019.
- SMR_B,j_ is the standardized mortality rate in geographical area B for the corresponding year j.
- SMR_RO,j_ is the standardized mortality rate in Romania, in year j, as it resulted from the national standardization.

RU MD_C,j_(%) = (SMR_R,C,j_ − SMR_U,C,j_)/SMR_RO,j_ (5)

where:

- RU MD_C,j_ is the rural–urban mortality difference in region C in year j, expressed as percentage of the national SMR.
- Area C could be any of the eight regions of the country.
- Year j could be 2001 or 2019.
- SMR_R,C,j_ is the rural standardized mortality rate in region C for the corresponding year j.
- SMR_U,C,j_ is the urban standardized mortality rate in region C for the corresponding year j.
- SMR_RO,j_ is the standardized mortality rate in Romania, in year j, as it resulted from the national standardization.

The MRs were expressed as old–recent value, and the MDs were expressed as subnational–national values. Thus, due to the decline over the years, all the MRs are positive, and the subnational areas with rates below the national have negative MDs, which is a favorable situation.

Ranking: In the subnational analysis per region, we ranked the regions from 1 to 8, where 1 = most favorable and 8 = least favorable. If two regions had the same position, they received the same ranking.

Chi-square test was used for comparing the changes in number of deaths, with a significance level of 95%.

## 3. Results

### 3.1. Differences between Romania and the EU

#### 3.1.1. Number of Deaths

In 2001, 10,570 deaths due to cervical cancer were registered in the EU, out of which 1763 (17%) were reported for Romania [15]. At that time, Romania had the third-highest number of deaths among the EU members, after Poland and Germany, and the three countries together accounted for 51% of EU deaths. In 2016, the annual number of deaths had decreased by 8% and 10% in the EU and Romania, respectively, compared to baseline (2001). In Germany and Poland, reductions of 11% and 13%, respectively, were revealed. Despite this reduction in deaths in Romania, the country still accounted for 15% of the EU cervix cancer deaths in 2016.

#### 3.1.2. Mortality Rates

Both Romania and the EU have shown decreasing trends in cervix cancer mortality during the study interval, but the rates for Romania were constantly much higher compared to the EU. In 2002, Romania had the highest cervix cancer mortality in the EU, and the mortality difference compared to EU was enormous (276%, meaning 18.8 versus 4.8 deaths per 100,000 women in Romania and EU, respectively; the year 2001 was not available for EU). The second-highest SMR was found in Lithuania, followed by Poland (16.4 and 10.9 deaths/100,000 women).

In 2016, the mortality reduction reached 58% and 56% for the EU and Romania, respectively, compared to 2002. However, the mortality difference remained unchanged (277%). Lithuania and Poland have shown a 61% and 59% reduction in mortality over the years, and the ranking among the EU members did not change.

### 3.2. Differences within Romania

#### 3.2.1. Number of Deaths

In 2019, Romania reported 1539 deaths, meaning a 13% decline compared to 2001. (A 10% decline was already reported for 2016.) In 2001, 51% came from rural areas, and this percentage changed significantly, to 46%, in 2019 (*p* = 0.013) (Table 1). A more important decline of deaths over the years was registered in rural areas (20% compared to 5% in urban areas).

In 2001, the NE region was the biggest contributor to the total deaths, followed closely by S and NW, and the lowest contributor was the BI region. In 2019, S became the highest contributor to the total deaths, followed by NE and SE. BI remained the lowest contributor, despite an 8% increase in the number of deaths. The S region is the only one (except BI) with an increase in the number of deaths in 2019 (8%). If the situation of Bucharest was caused by an increase in population, due to the fact that this is the most developed region of the country, the S region probably had other determinants for this increase. In fact, significant changes in contribution to the total deaths occurred in only three regions: S (increase) and NE and SE (decrease) (Table 1). Overall, Romania, for the study interval (2001–2019), reported 32,558 cumulated deaths due to cervix cancer, out of which 47% occurred in the rural areas. Additionally, 61% of these deaths were registered in adult women (aged 0–64 years) (Table 2).

#### 3.2.2. Trends in Cervix Cancer Mortality

**Figure 2:**
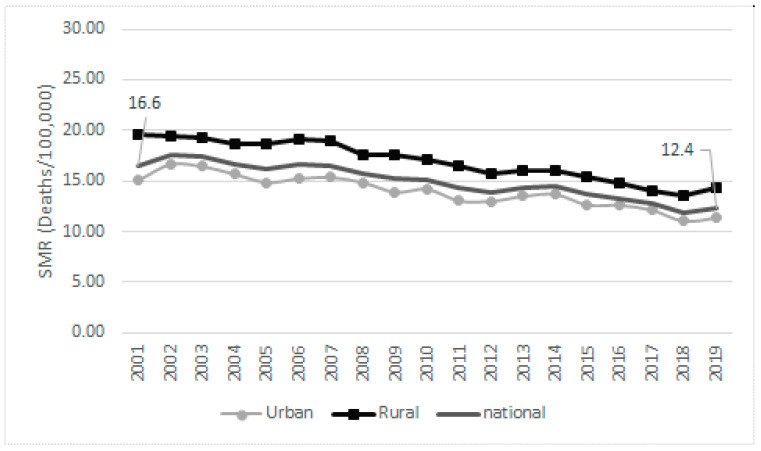
Trends in cervix cancer mortality, overall and by rural–urban areas, Romania, 2001–2019 (standardized mortality rates).

**Figure 3:**
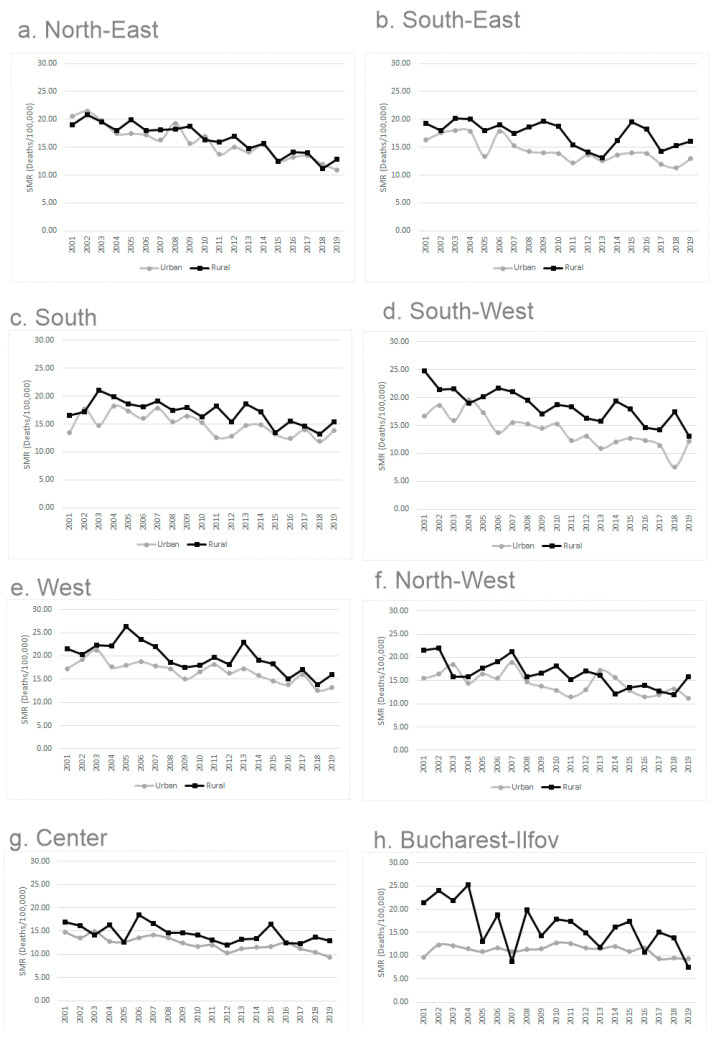
Regional trend in cervix cancer mortality by rural–urban areas, Romania, 2001–2019 (standardized mortality rates).

The standardized mortality rate at the national level revealed a decreasing trend during the study period, but it was constantly higher in rural areas (Figure 2). The declining trend was seen at different extents in all the regions, but with the same pattern of higher mortality in the rural population (Figure 3).

#### 3.2.3. Mortality Reduction over the Two Decades

Overall, the mortality reduction reached 25% in 2019 compared to 2001 for the national rate and 22% and 32% for the urban and rural rates, respectively (Table 3). In the regions, the most important reduction was found for the SW and NE, reaching 44% and 43% decline compared to baseline, but important reductions also occurred in NW, C, and W (Table 3). More limited progress was found in the SE (18%) and Bucharest (8%); meanwhile, in the S region, the situation remained almost unchanged compared to 2001 (Table 3).

The analysis of rural and urban models of mortality by regions revealed a remarkable progress in improving the rural models of mortality in BI and SW regions, with an 84% and 71% reduction in rural mortality. To a moderate extent, this improvement also occurred in the NE, NW, and W (MR of 37%, 35%, and 33% in rural). The most modest improvement in rural mortality was noticed in the S (only 7%) (Table 3). The urban models have shown a marked progress in NE (58% MR) and a moderate one in the C, NW, and W (32%, 26%, and 25%, respectively); meanwhile, the MR was modest in BI (3%) and negative in the S (−2%) (Table 3). Overall, in five regions, the MR was more marked for rural (BI, SW, NW, W, and S); in the other two, it was more marked for urban areas (NE and C), and in the SE, the urban and rural improvements were similar.

#### 3.2.4. Past and Current Differences in Mortality

In 2001, the most detrimental regional MDs were seen in the SW, NE, and W (19%, 14%, and 12% difference compared to the national model) and most favorable in BI, the S, and C (−37%, −11%, and −9%, respectively) (Table 4). The highest RU-MDs were seen in BI, the SW, and NW (40%, 49%, and 37%, respectively) and the lowest ones in the NE and C (−9% and 13%, respectively, the N being the only region in which rural mortality is lower than the urban one) (Table 4). At the national level, the RU MD reached 27%.

In 2019, the W, S, and SE regions showed the highest MDs, and BI and C were in the best positions. Regarding the RU MD per region, it was most marked in the NW, C, and SE (37%, 28%, and 25%, respectively), and the lowest in BI and the SW (Table 4). For the national level, the RU MD reached 24%

## 4. Discussion

Our research revealed important differences in cervical cancer mortality between Romania and the EU and within Romania. These differences were very high in the past and, despite a 13% reduction in deaths and 25% reduction in mortality over the two decades, they are still persisting. At the EU level, Romania is keeping the first position in mortality rate and shows a huge difference compared to the EU’s rate. Within the country, the rural population continues to remain more disadvantaged. Despite a higher reduction in rural mortality, compared to the urban one, the rural–urban mortality gap still achieved 24% of the national rate in 2019.

The analysis per region also reflects the different rhythms of progress in mortality reduction, persisting gaps compared to the national level, and, in some cases, major gaps between rural and urban populations. For example, the SW, NE, and W had the highest deviations compared to the national model in 2001, but meanwhile, the SW and NE succeeded to achieve good progress (44% and 43% MR), while the W region reached only a moderate one (27% MR). The poorest transformation was seen in the S region, which started with a favorable MD compared to the national model, but it achieved a very limited gain only in rural mortality. Another particular situation is related to the NW region, which has a very important tradition in implementing cervical cancer screening programs [12]. Despite this wide experience, the progress in the region was moderate, with a 33% mortality reduction over the two decades, a persistent mortality difference of 5% compared to the national model, and a constant rural–urban difference of 37% in favor of urban. The BI region had from far the most privileged situation in 2001 (37% mortality difference compared to the national model), but with the widest rural–urban gap in the country (70%). This gap has been corrected over the years, but it is uncertain if correction occurred due to mechanisms for improving access to services for the rural population or to the socio-economic particularities of the region. This region includes Bucharest (the capital city) and the county of Ilfov, with approximately one-fifth of the Bucharest population. The city of Bucharest is geographically surrounded by Ilfov, and the only rural population of the region (9%) belongs to the county of Ilfov. During the last two decades, the people mobility between Bucharest and Ilfov increased substantially, on the background of the economic development. This mobility could explain the yearly variation of mortality line in the rural population of Ilfov.

Our results underline the persisting barriers of access to preventive and curative services for the rural population, despite the fact that the legislative framework guarantees equitable access to services for all citizens. The health system in Romania is social-insurance-based, the health insurance is compulsory, and the system is defined to ensure universal access to primary care and referral-based specialized care [26]. For equity reasons, many vulnerable categories of people are insured without payment of financial contribution [26]. More than this, certain national health programs complement the package of services provided by health insurance, among which the oncology program (ensuring free access to cytostatic treatment for all oncologic patients, including the case of cervical cancer) and the cervix cancer screening program (ensuring free access to pap-smear screening for all insured and non-insured women) [27]. These programs, and in particular the cervix cancer screening, are, in theory, accessible for all, but the participation remains weak (5% achievement of the annual target for cervix cancer screening in 2018) [28]. In general, cancer diagnostic and treatment are available and fully covered by the insurance package (clinical procedures) and the national health program (cytostatic), being available for insured and non-insured women. The access to services is based on a referral from the family doctor or direct, in case of emergency (through the emergency department). Once diagnosed, there is direct access to all specialized services (surgical treatment, cytostatic, and/or radiotherapy).

On the contrary, the preventive services are fewer, less accessible, and partially or not covered by either the health insurance or the national programs, despite consistent efforts to develop them over the years. For example, the first attempt to introduce a free HPV vaccination was made in 2008, as a state-financed program targeting young girls (10–11 years old), but that program failed with only 2.5% of the eligible population being vaccinated due to the huge hesitancy of the parents [29]. Following this failure, the program has been changed, with the free vaccine provided based on request for girls aged 11 to 18 years, but the demand remains very limited. Boys and women older than 18 years of age were and are not targeted by the free vaccination program. For these categories of people, the vaccine was available in pharmacies, prescription-based, and with full out-of-pocket payment. In these circumstances, the HPV vaccination uptake remained very limited. Complementary, HPV testing is available in the country at full price (not included in the package of services covered by health insurance). This testing has been provided for free in some projects and revealed a high-risk HPV prevalence of 18% among women 18 to 70 years old [30]. Regarding early diagnosis, colposcopy is available in most outpatient specialized clinics and hospitals and covered by insurance in some circumstances, but the providers are located usually in cities, and women from rural or deprived areas have limited access due to geographic, information, and, sometimes, financial barriers [26]. No audit mechanisms have been implemented in relation to cervical cancer prevention services.

A major question is what actions should be taken to improve cervical cancer control in the future. There is obviously a prioritization of cervical cancer screening in the country. The national screening program has been introduced for many years, despite its failure in ensuring a population-based screening and appropriate participation [28]. Evidence for good practice models is available in the country and could serve as a basis for revisiting and improving the existing implementation of the national program [31,32,33,34,35,36,37]. Certain interventions are being planned, such as a project for the integration of primary HPV screening into the national cervical cancer screening program [36]. This project is planned to be implemented in two phases: a training and planning phase, which is in ongoing implementation, and the screening itself, which is planned to be implemented in the North-West, Center, South, and North-East, having at least 170,000 screened beneficiaries per region [38].

Our results provide the appropriate framework for understanding the evolution of cervical cancer mortality over decades, the differences within regions, and the rural–urban gaps. As a consequence, the national program should be remodeled, by focusing on the rural population and, in particular, in the West, South, and South-West regions, which still kept a major difference in mortality compared to the national level. Another essential premise for the national program is to ensure the population-based character of the screening, which is in line with the European guidelines, and with the Romanian law, which guarantees universal access to preventive services for all the citizens [9,10,11,14,26]. Despite being reported as a population-based program, due to the modest participation rate, the programs should be analyzed in their main stages, and more feasible remedial mechanisms should be identified.

Another important aspect is the active character of the program, with individual invitations through the screening registry or database (which does not exist in Romania), a fixed appointment date included in the invitation, and a continuum of services for positive cases [12]. Last but not least, dedicated human and financial resources and procedures and clear quality-assurance mechanisms should be analyzed and implemented [12].

Of course, the situation of Romania is not singular, despite its huge gap in cervical cancer mortality compared to the EU. Evidence suggests that cervical cancer mortality rates are higher in Central-Eastern Europe, and the access to organized screening programs is more detrimental compared to the rest of the continent [12,39,40,41]. All these facts are against the evidence that women attending organized screening programs versus non-attenders could benefit from a 41% to 92% reduction in cervix cancer mortality, according to a recent systematic review [42]. These facts could be considered at the EU level for building more accessible opportunities and exchanges related to cervical cancer screening as a feasible way to decrease the gaps in mortality among the EU member states.

## 5. Conclusions

In conclusion, cervical cancer represents an important public health problem in Romania, despite its declining number of deaths and mortality rate, over the last two decades. Important differences are persisting between Romania and the EU and within the country, on the background of the failure in organizing a population-based national screening program. The country should use the existing opportunities to improve its screening program in the upcoming years. Due to the similar problems existing in Central-Eastern Europe, targeted support from the EU for the members from this geographical area could contribute to the minimization of differences in cervical cancer mortality among the EU members.

## Figures and Tables

**Table 1:** Deaths by cervix cancer in Romania, 2001 and 2019, national and subnational distribution and percentage change.

Sub-National Area	2001			2019			*p*-Value *	% Change in 2019
	Number	%	Rank	Number	%	Rank		
National	1763	100%	NA	1539	100%	NA	NA	13%
Urban	870	49%	NA	825	54%	NA	0.015	5%
Rural	893	51%	NA	714	46%	NA		20%
**Regions**								
North-East	316	18%	1	235	15%	2	0.041	26%
South-East	237	13%	3	224	15%	2	0.358	5%
South	246	14%	2	265	17%	1	0.009	−8%
South-West	228	13%	3	156	10%	5	0.012	32%
West	182	10%	4	165	11%	4	0.709	9%
North-West	243	14%	2	203	13%	3	0.619	16%
Center	184	10%	4	154	10%	5	0.684	16%
Bucharest–Ilfov	127	7%	5	137	9%	6	0.072	−8%

* Chi-square test; NA-not available

**Table 2:** Cumulated deaths by age groups and rural–urban areas, 2001–2019.

Age Group	Urban		Rural		National	
	No.	%	No.	%	No.	%
0–44 years	2251	13%	2318	15%	4569	14%
45–64 years	8771	51%	6577	42%	15,348	47%
65+ years	6022	35%	6619	43%	12,641	39%
Total	17,044	100%	15,514	100%	32,558	100%
% of national deaths	53%		47%		100%	

**Table 3:** Mortality reduction over decades—national, regional, and urban–rural.

Type	National	NE	SE	S	SW	W	NW	C	BI
National	25%	43%	19%	2%	44%	27%	33%	28%	8%
Urban	22%	58%	20%	−2%	27%	25%	26%	32%	3%
Rural	32%	37%	20%	7%	71%	33%	35%	24%	84%

**Table 4:** Mortality difference by region and urban–rural areas.

	2001				2019			
Region	Regional MD	Rank	RU MD per Region	Rank	Regional MD	Rank	RU MD per Region	Rank
North-East	14%	7	−9%	1	−5%	3	16%	4
South-East	3%	4	18%	4	12%	6	25%	6
South	−11%	2	18%	3	17%	7	12%	3
South-West	19%	8	49%	7	0%	4	7%	2
West	12%	6	26%	5	14%	8	23%	5
North-West	11%	5	37%	6	5%	5	37%	8
Center	−9%	3	13%	2	−15%	2	28%	7
Bucharest–Ilfov	−37%	1	70%	8	−26%	1	−15%	1

## Data Availability

The datasets used and analyzed during the current study are available from Eurostat and the National Institute of Statistics, Bucharest, Romania, on reasonable request.
